# Conducting a randomised controlled trial of a psychosocial intervention for adolescents with type 1 diabetes during COVID-19: recommendations to overcome the challenges complicated by inconsistent public health guidelines on research

**DOI:** 10.1186/s13063-022-06314-9

**Published:** 2022-04-27

**Authors:** Sinead Pembroke, Shauna Rogerson, Imelda Coyne

**Affiliations:** Trinity College Dublin, College Green, Dublin 2, Dublin, Ireland

**Keywords:** COVID-19, Pandemic, Randomised control trial, Psychosocial intervention, Trial management

## Abstract

Since the beginning of the COVID-19 pandemic, there has been very little guidance in Ireland and abroad, around the conduct of research, and randomised controlled trials (RCTs) in particular. This has led to inconsistent interpretations of public health guidelines for the conduct of research in hospitals. Consequently, challenges have arisen for researchers conducting RCTs, in relation to recruitment and retention. These challenges are amplified for RCTs of psychosocial interventions, where communication and physical contact play a major role in administering the RCT. Therefore, learning from other research studies is important. This study addresses the challenges in administering an RCT of a psychosocial intervention in two paediatric outpatient diabetes clinics in Dublin Ireland, including recommendations to overcome these. Recommendations include the following: (1) recognise research as an essential service; (2) hospital management should implement guidelines to ensure a consistent approach to the conduct of research during pandemics; (3) ensure that there is a mechanism for the provision of clear and effective communication before the clinic visit with patients, to reassure them and gain their trust; and (4) trial managers should make time to check in with their team every day, as they would do if they were in the office.

## Background

Since the beginning of the COVID-19 pandemic, there has been a paucity of guidance in Ireland and abroad, around conducting randomised controlled trials (RCTs) [1, 2]. Even though regulatory authorities have provided guidance on running clinical trials during the pandemic (for example, the European Medical Agency (EMA) [3] and the Health Products Regulatory Authority (HPRA) [4]), there have been inconsistencies in government guidance around the essential nature of research. This has had a tumultuous impact on research conducted in hospitals, as advice has continually changed over the course of the last three national lockdowns in Ireland (March 2020 to January 2021), where access has been granted and then later denied to researchers during similar periods of restrictions.

One key question that remains ambiguous is, is research an essential service? [1] Many authors would suggest that research which produces new knowledge and reduces uncertainty is essential [5, 6]. However, this has not been given consideration under public health guidelines in Ireland. The government imposed restrictions have created many obstacles, and this has been particularly challenging for RCTs involving direct patient contact in hospitals.

Literature continues to emerge on the experience of conducting research during the COVID-19 pandemic. Valuable insight has been contributed by authors giving their accounts of running clinical trials [7, 8] and the problems they encountered [6, 9]. Previous studies have explored the role of the clinical trial manager and guidance for future clinical trials [1]. Other papers have looked at the role of technology in overcoming the challenges of the COVID-19 pandemic [10]. Furthermore, a systematic review highlighted the need for more support to produce high-quality research and to construct a collaboration sharing platform to prepare and support research teams during a pandemic [11]. Sharing expertise and learning from other research studies who are in a similar situation is important [6], especially where there is little or varying public health guidelines on continuing research during a pandemic [1]. Although there are numerous papers reporting on managing clinical trials during the COVID-19 pandemic, there is very little addressing the challenges of conducting an RCT of a psychosocial intervention, which requires direct patient contact in a hospital setting [12, 13].

This paper contributes to the literature on the COVID-19 pandemic, with a focus on our experience of administering a face-to-face trial of a psychosocial intervention in two paediatric outpatient clinics in Dublin, Ireland. Our paper discusses the challenges involved during the COVID-19 pandemic amidst the various levels of restrictions in place, and the most effective solutions for adapting to public health guidelines, during a time of inconsistency and uncertainty, particularly for research. Finally, recommendations are suggested, which can be considered for future research/trials.

### The ‘Promoting Adolescent Communication and Engagement’ (PACE) study

This study aimed to develop and evaluate an intervention to improve adolescent question-asking and provider education during paediatric diabetes visits. The study began in January 2019 and included three phases. The first phase comprised focus groups and one-to-one interviews with adolescents, caregivers, and healthcare providers, which served to inform the content of an educational video and question prompt list [14]. Phase two consisted of the design and development of an educational video, highlighting the importance of adolescents’ active involvement in communication exchange during diabetes visits, and a question prompt list to enable adolescents to ask their questions of healthcare professionals. Phase three involved conducting an RCT to compare the effectiveness of the intervention with usual care in two paediatrics diabetes clinics.

Phases one and two were completed by March 2020, when the first lockdown due to COVID-19 started. The third phase (the RCT) started in October 2019 in two paediatric diabetes clinics in tertiary children’s hospitals in Ireland. Adolescents’ eligibility criteria included (1) aged 11–16 years, (2) diagnosed with type 1 diabetes, (3) previously visited the diabetes clinic at least once, (4) no additional medical diagnosis and (5) spoke English. The target recruitment number for the RCT was 140 adolescents and parents (dyads). The intervention (video and QPL) was to be administered at three time points, which were due to occur between October 2019 and June 2021.

Adolescents who passed the eligibility criteria were recruited. Eligibility was screened by the clinics’ data managers, who posted out an information sheet explaining the study to eligible adolescents at least 1 week before their visit. These patients were approached by the researcher in the waiting area of the paediatric diabetes clinic. If the adolescent and their caregiver were happy to participate, they were consented for the study and allocated to either the intervention or control group. All participants were asked to partake at three time points in the study.

**Fig. 1:**
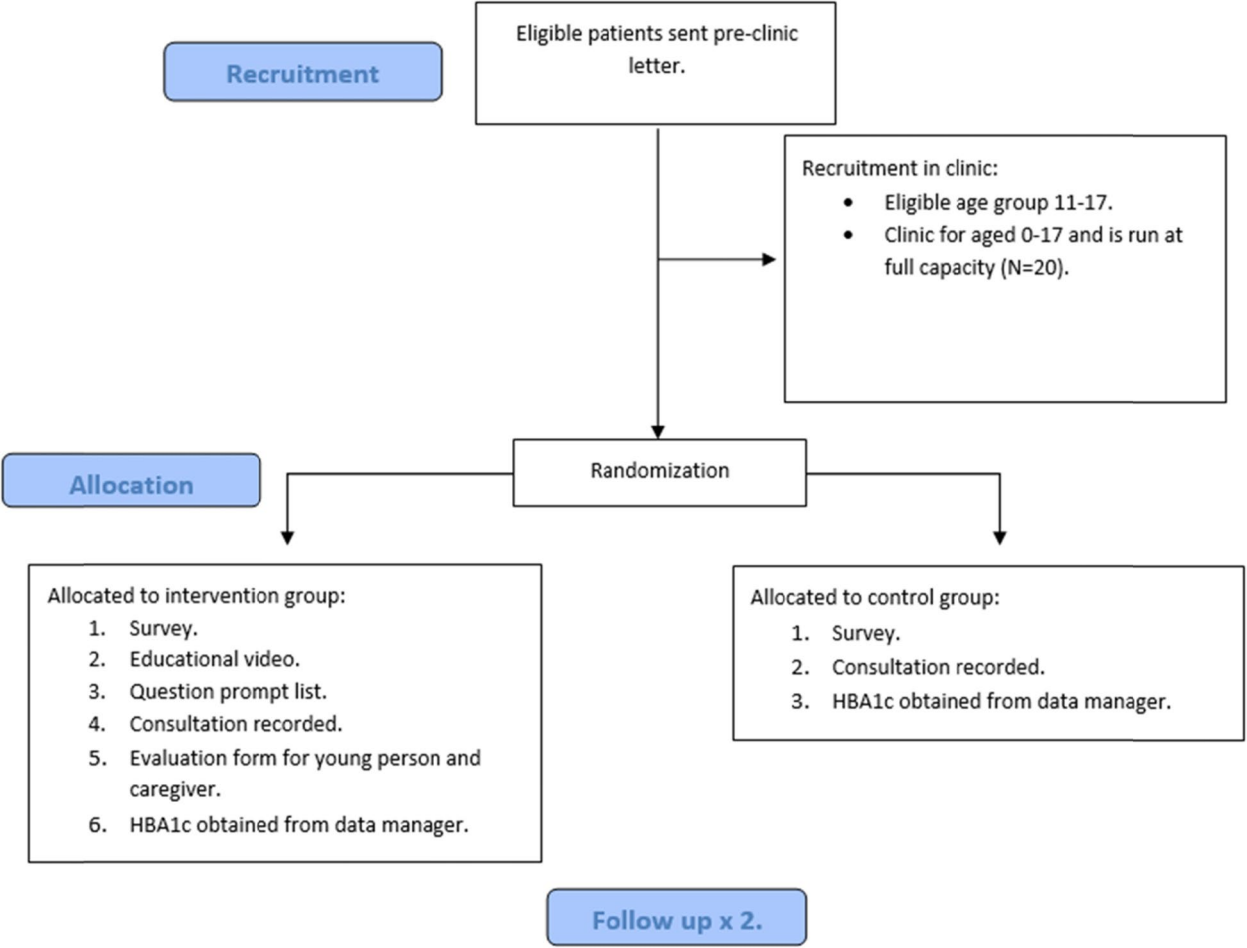
Flow diagram of the recruitment process

In both intervention and control groups, patients completed a 15-min survey, their HbA1c (average blood glucose level over 3 months) was recorded, and the doctor’s consultation was audio recorded. For the intervention group, in addition to the above, they also watched the video on a tablet with headphones provided by the researcher, which was just under ten minutes long, and completed the Question Prompt List. The intervention group also completed a short evaluation form on the intervention at the end of the consultation (please see Fig. 1). The time to administer the RCT for the control group took approximately 15 to 20 min, and for the intervention group approximately 30–40 min.

### Challenges and solutions in trial management during the pandemic

#### Challenge 1: lack of consistency in the designation of research as essential

Following the first detected cases of COVID-19, Ireland went into a national lockdown in March 2020. As a result, data collection in the two hospital sites was temporarily paused as research was deemed to be non-essential, and access was denied.

Following the lifting of hospital restrictions and considerable negotiation with the diabetes teams in both hospitals, the research team resumed data collection in June/July 2020 (one hospital allowed access sooner than the other).

**Fig. 2:**
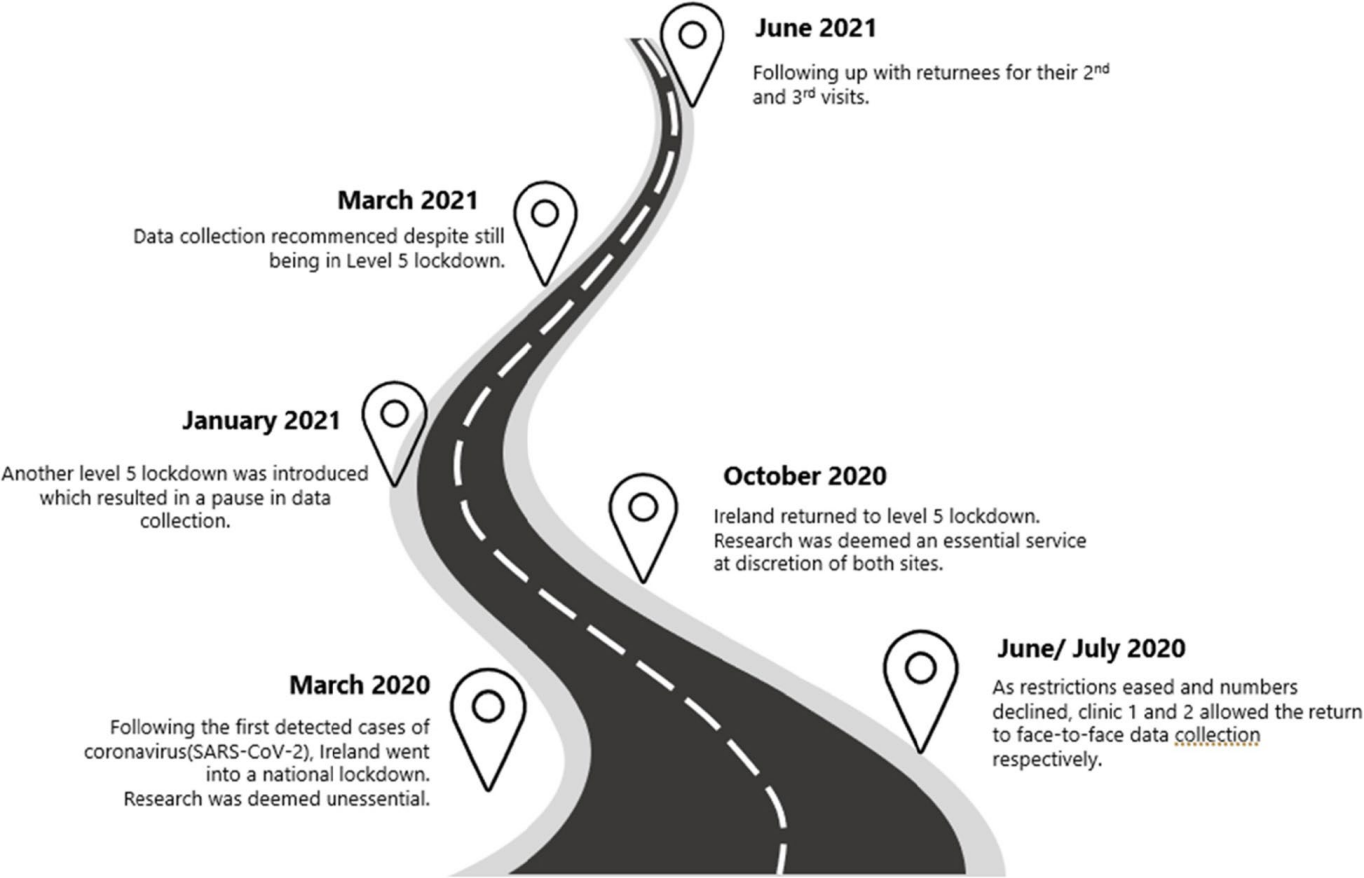
RCT data collection road map from March 2020 to June 2021

In September 2020 the Irish government introduced a ‘National Framework for living with COVID-19’ (https://assets.gov.ie) that outlined 5 levels of restrictions, with level 5 being the most restrictive. In October 2020 due to rising COVID cases, there was a return to level 5 restrictions. At that point, the research team could continue to have direct access to participants in the hospital diabetes clinics, despite it being level 5 restrictions across the country. However, after a short period when restrictions were loosened, the government re-imposed level 5 restrictions again in January 2021, (this was a unique lockdown in Ireland due to case numbers being exceptionally high). During this time, the research team were restricted from accessing the hospital and had to stop data collection again for 3 months (see Fig. 2). Data collection resumed in March 2021 despite level 5 restrictions in place.

Consequently, since March 2020, when public health restrictions were put in place, there appeared to be considerable inconsistency in relation to designating research as essential. This had a major impact on the trial itself, particularly recruitment and retention. It was left up to hospitals and departments within hospitals to decide whether to allow researchers access rather than a clear policy being put in place. The trial funding and timescale remained the same, yet 7 out of 17 months allocated to recruitment were lost. Furthermore, these two periods of lockdown meant that patients were missed returning for their second and third data collection time points. In some instances, this delay led to patients graduating into the adult services and thus they could no longer participate in the trial.

#### Solution: an active line of communication between the trial manager and the healthcare team

Maintaining an active line of communication between the trial manager and the healthcare team in both sites was essential as it encouraged support for data collection to continue throughout the pandemic. This was also identified in other studies reporting on clinical research during COVID-19 [1, 2]. Virtual meetings between the project manager and members of the diabetes team in both clinics, (including administrators, advanced nurse specialist and consultant endocrinologists) took place during the first lockdown in March 2020. The research manager also attended the two clinical sites to plan for the return of data collection in both sites. From these consultations, a revised protocol was established, detailing how the RCT would be conducted once researchers were allowed access to the hospital sites again. As it was the healthcare team’s decision to allow researchers access to the clinic, the protocol helped instil confidence that the return of research staff could be done safely. Agility, quick thinking and continuous review of different scenarios that arose were imperative to adapt and overcome obstacles, ensuring that the RCT could continue without negatively impacting on the safety and care of participants [1].

#### Challenge 2: recruitment, retention and the unpredictability of appointment scheduling

When Ireland went into lockdown in March 2020, hospital outpatient clinics moved to virtual appointments for all patients. Following the easing of restrictions in June/July 2020, face-to-face appointments were re-introduced, but virtual appointments remained, accounting for at least half of all appointments throughout 2020 in both paediatric diabetes clinics.

The COVID-19 pandemic saw a large shift to virtual technology globally. This was also the case for hospitals in Ireland, which saw many outpatient clinics moving to virtual clinics. Positive reports for telehealth have been reported within the literature, citing improvement in communication with patients and saving time for healthcare professionals and patients alike [10]. However, this is contrary to the experience of both diabetes clinics, where reports from healthcare professionals suggested a decrease in communication with adolescent patients, as they were not present or, if they were present remained silent on the phone. Since the engagement of the young person was the aim of this study, a decision was made between the research team and healthcare providers not to include virtual appointments.

Adolescents attending the diabetes clinics usually rotated from face-to-face to virtual clinics, with the cycle between visits averaging from 16 to 21 weeks. The research team had no input or control in scheduling appointments. If a patient enrolled in the RCT received a virtual appointment, this meant that the research team would have to wait until the next face-to-face appointment. Furthermore, even when scheduled for a face-to-face appointment, patients had the option to switch to a virtual appointment if they preferred.

This added a layer of uncertainty for the research team when it came to follow-ups at times two and three of the RCT. As the RCT required collecting data in person at three time points, the combination of virtual and face-to-face appointments at each diabetes clinic made it challenging for this to be completed within the project timeline. This is because if the adolescent was scheduled for a virtual appointment, the next appointment would not be due again for at least 3 or 4 months.

Furthermore, the newly implemented health and safety guidelines within the hospitals meant that the waiting time to see healthcare providers in the diabetes clinic decreased significantly. In line with health and safety protocols, the clinics avoided having patients waiting for long periods, as waiting area spaces were significantly reduced due to social distancing requirement. Before the pandemic, adolescents might have to wait between one to two hours, which allowed ample time to carry out the RCT.

With clinics operating at half capacity, adolescents were seen much quicker, sometimes within minutes of arriving. Occasionally, it happened that the doctor called the adolescent for their consultation, while the researcher was in the process of recruiting or administering the RCT. This resulted in the loss of potential recruits and missing follow-up data. Even though the healthcare providers in both clinics were very supportive, they felt that it was imperative that the trial did not interfere in the running of the clinic, especially given that a delay could have serious consequences for the health and safety of all at the clinic. Altogether, these challenges impacted recruitment, and consequently, this resulted in a smaller sample than originally planned.

#### Solution: adapt the protocol with support from the hospital healthcare teams

To overcome these challenges, the trial manager adapted the protocol so that the data manager in each clinic would send out pre-clinic letters with a copy of the survey to complete at least 1 week before their appointment. For those in the intervention group, this also included a link to the video, and they were asked to watch it before their appointment. For recruitment of eligible patients, information sheets were already being sent out before the pandemic by the data manager at least 1 week before their appointment. The information sheet was altered to include a line asking the parent to contact the research team directly before attending the clinic if their son/daughter was interested in participating. This way, the research team could reduce physical contact by discussing the study, answering any questions, and administering the survey before the appointment date.

Working with members of the healthcare team on these changes was important, particularly data managers who manage patient appointments. Gaining assistance from the data managers in each site who could access patient details and screen for eligibility enabled compliance with data protection regulation and allowed the research team to overcome obstacles imposed by the COVID-19 pandemic. Debriefing conversations between the research team, clinicians and data managers after clinics provided opportunities to discuss what worked, what was challenging, and what could be done differently. These conversations informed the adaptations made throughout the RCT.

#### Challenge 3: interacting with patients during the COVID-19 pandemic

This study required face-to-face communication and interaction with the adolescent and their caregiver. For example, communication was central for recruiting adolescents, to administer the intervention, and the survey. The pandemic and the public health measures created challenges for communicating with adolescents and administering the RCT in several ways. First, it was evident that some adolescents and caregivers were very anxious about their health and safety and, consequently they reported feeling very uneasy upon returning to clinic. This was particularly the case in the early months of the pandemic, and directly after long periods of level 5 restrictions. Second, the lack of additional space in one hospital site meant that the RCT was administered in an often busy waiting area with a limit on the maximum capacity as part of its health and safety protocol. The limited space posed difficulty for recruiting potential participants, as the researcher required time and space to explain the study, answer any questions and gain consent. If the clinic reached maximum capacity, the researcher was required to leave the waiting area, and potential recruits were lost.

#### Solution: increase pre-clinic communication with patients

During a global pandemic, trust is an important consideration when deciding whether to take part in clinical research [15]. Therefore, increased communication with potential recruits and adolescents and parents enrolled in the trial helped maintain confidence in the research team. Increased stress and anxiety in relation to risk of infection and transmission [16] meant that written communication in the form of pre-clinic letters, was a chance to reassure participants of the safety procedures the team had put in place. In the second hospital site, an arrangement was made to secure an office where the RCT could be administered. This was significant, as from a health and safety perspective, it also made the participants feel more secure in taking part. These measures helped to decrease contact with participants and ease any health and safety anxieties they might have. This had a positive effect on retention, as no one refused to continue their participation in the trial.

#### Challenge 4: boosting the research team’s morale while working remotely

With the closure of all services abruptly in one day in March 2020, basically overnight the research team had to switch from being together in an office and having regular and informal face-to-face meetings, to remote working and virtual meetings instead. As the research team worked remotely in their homes and at separate hospital sites, team morale required extra attention, especially for forging connections, providing emotional support, and facilitating effective communication between the project manager and the team.

#### Solution: maintain regular communication with the team without overdoing it

The project manager made regular check-ins by email and phone to individual members of the team. For group meetings between the whole team, this was facilitated by virtual meetings. With everyone more dependent on technology than ever before, the challenge was balancing supportive contact while also considering and respecting researchers’ competing demands and time needed to disconnect from technology. Recently coined term ‘zoom fatigue’ illustrates the increased mental exertion required for online interactions [17], which can lead to emotional exhaustion. This was something to be mindful of, and extra precautions were taken not to organise more virtual meetings than we would have had in person.

## Conclusion

We would like to suggest four recommendations for future conduct of RCTs during a pandemic. Firstly, one of the main challenges was that research was not designated by the Irish government as essential [1]. Consequently, decisions were taken at a local level on giving access to the research team to the hospital sites. Therefore, the first recommendation is to recognise research as an essential service. Not only would this be a recognition of the important role that research plays in society, but it would provide certainty for future periods of restrictions.

Even when the research team were given access to both hospitals, challenges were still faced, particularly in relation to the recruitment and retention of participants. Therefore, a second recommendation is for hospital management to take a leading supportive role and facilitate the research team in whatever way they can to ensure the research can continue. Other studies also conclude that collaboration between the clinic and the research team is essential during a pandemic [5]. Our study benefitted from having two healthcare teams that supported our revised trial protocol and provided resources, (sending out information sheets to participants and potential recruits and providing a room to conduct the trial). Despite the myriad of healthcare challenges and stress imposed by the pandemic on healthcare staff, they were very helpful in working with us to identify ways to overcome challenges in the conduct of our research study.

A third challenge was communicating with participants at a time when there was a lot of anxiety around social interactions with other people. This was particularly problematic for our trial given the interactive format of the intervention. A third recommendation is to ensure that there is a mechanism for the provision of clear and effective communication before the clinic visit with patients, to reassure them and gain their trust. This is particularly important for trials involving direct patient contact.

Finally, the research team’s morale is an important consideration for any trial manager. A fourth recommendation is for trial managers to make time to check in with their team every day, as they would do if they were in the office. However, trial managers should ensure that they do not organise excessive online meetings to avoid virtual meeting fatigue [17].

We were not alone in the challenges we encountered as other trials also faced a similar fate [1, 9, 18]. Other research studies and RCTs also faced similar problems, including having to stop the trial as participants had a health condition that made them particularly vulnerable to COVID-19 [18], challenges with ethics applications for amendments to research protocols [6], and a lack of guidelines on conducting RCTs during a pandemic [1, 11]. However, what made it even more problematic was that we were trialling a psychosocial intervention, which centred on increasing communication and engagement among adolescents. Thus, administering the RCT also involved a lot of direct face-to-face contact and communication with patients. Interestingly, some RCTs testing out psychosocial interventions did not experience similar challenges because they were able to recruit virtual patients, whereas we were not allowed to do so [12, 13].

These were some of our challenges associated with managing a clinical trial during an unprecedented major pandemic crisis without time to prepare, high levels of fears and uncertainty and lack of clear policies on research. Restrictions have now largely been lifted. However, the challenges described are not in the past; anxiety concerning COVID-19 still exists for some and many want to limit social interactions. Virtual appointments still feature in healthcare settings. Furthermore, COVID-19 is still creating a volatile situation in hospitals in Ireland, and it is possible that restrictions in healthcare settings could return. While the COVID-19 pandemic was unprecedented, we can learn from it, and it is important that we are prepared if the situation arises again. This article contributes to that learning by making recommendations when conducting RCTs during a pandemic, particularly RCTs that involve direct patient participation. Hopefully, these experiences may be informative for researchers facing similar challenges in future scenarios.

## Data Availability

Not applicable.
